# Retraction: A predictive model based on ground glass nodule features via high-resolution CT for identifying invasiveness of lung adenocarcinoma

**DOI:** 10.3389/fsurg.2023.1307339

**Published:** 2023-10-13

**Authors:** Frontiers Editorial Office

A Retraction of the Original Research Article A predictive model based on ground glass nodule features via high-resolution CT for identifying invasiveness of lung adenocarcinoma By Yan B, Chang Y, Jiang Y, Liu Y, Yuan J and Li R. (2022). Front. Surg. 9:973523. doi: 10.3389/fsurg.2022.973523

The journal and Chief Editors retract the 26 August 2022 article cited above.

This article is retracted by Frontiers. The publisher has discovered that the authors provided false information for the peer-review process. As the scientific integrity of the article cannot be guaranteed, and adhering to the recommendations of the Committee on Publication Ethics (COPE), the publisher therefore retracts the article.

The authors have not responded to this retraction.

This retraction was approved by the Chief Editors of Frontiers in Surgery and the Chief Executive Editor of Frontiers.

